# The Key Role of IP_6_K: A Novel Target for Anticancer Treatments?

**DOI:** 10.3390/molecules25194401

**Published:** 2020-09-25

**Authors:** Mirko Minini, Alice Senni, Vittorio Unfer, Mariano Bizzarri

**Affiliations:** 1 Department of Experimental Medicine, Sapienza University of Rome, 00161 Rome, Italy; alice.senni180595@gmail.com; 2 Department of Surgery ‘P. Valdoni’, Sapienza University of Rome, 00161 Rome, Italy; 3 Systems Biology Group Lab, Sapienza University of Rome, 00185 Rome, Italy; vunfer@gmail.com

**Keywords:** inositol pyrophosphates (PP-IPs), diphosphoinositol pentakisphosphate (5-IP_7_ or IP_7_), inositol hexakisphosphate kinase (IP_6_K), *myo*-inositol, anticancer activity

## Abstract

Inositol and its phosphate metabolites play a pivotal role in several biochemical pathways and gene expression regulation: inositol pyrophosphates (PP-IPs) have been increasingly appreciated as key signaling modulators. Fluctuations in their intracellular levels hugely impact the transfer of phosphates and the phosphorylation status of several target proteins. Pharmacological modulation of the proteins associated with PP-IP activities has proved to be beneficial in various pathological settings. IP_7_ has been extensively studied and found to play a key role in pathways associated with PP-IP activities. Three inositol hexakisphosphate kinase (IP_6_K) isoforms regulate IP_7_ synthesis in mammals. Genomic deletion or enzymic inhibition of IP_6_K1 has been shown to reduce cell invasiveness and migration capacity, protecting against chemical-induced carcinogenesis. IP_6_K1 could therefore be a useful target in anticancer treatment. Here, we summarize the current understanding that established IP_6_K1 and the other IP_6_K isoforms as possible targets for cancer therapy. However, it will be necessary to determine whether pharmacological inhibition of IP_6_K is safe enough to begin clinical study. The development of safe and selective inhibitors of IP_6_K isoforms is required to minimize undesirable effects.

## 1. Introduction

Inositol is a ubiquitous polyol involved in a number of essential processes in living organisms. *Myo*-inositol is physiologically the most important of nine isomers and is the precursor of a bewildering number of complex inositol-containing molecules, including inositol phosphates [1,2]. Inositol compounds are essential for many biological functions in living cells: membrane biogenesis [3], trafficking [4], signal transduction, and regulation of gene expression [5]. Inositol phosphates are prominent mediators of these processes. Inositol-1,4,5-trisphosphate (IP_3_) has been widely investigated as an intracellular second messenger [6,7,8]. It is metabolized to a large number of additional inositol polyphosphates that also function as cell signals [9]. Among these, inositol hexakisphosphate (IP_6_), also known as phytic acid, is the most abundant inositol polyphosphate found in eukaryotes, identified as the principal phosphate-storage molecule in plant seeds [10,11]. It is involved in regulation of trafficking [12] as well as in several nuclear events [13,14]. Inositol hexakisphosphate is the building block to which successive phosphate groups are added to yield inositol pyrophosphates (PP-IPs) [15,16], where as many as one or two energetic di(β)phosphates bonds are crammed around the six-carbon inositol ring [17]. This class of molecule recently gained appreciation as critical modulators of a huge number of “signaling” pathways [18,19]. As proof of concept, PP-IPs show high turnover as their intracellular levels fluctuate significantly in various pathological disorders, including cancer [20].

## 2. Inositol Pyrophosphates

Inositol pyrophosphates have a di(β)phosphate group on their myo-inositol head. Several studies have unveiled many basic biological functions of IPs in mammals, including cell signaling [21], apoptosis [22,23], trafficking, cytoskeletal dynamics, autophagy, DNA repair, telomere maintenance, and insulin secretion [18,24]. Recent discoveries also indicate inositol pyrophosphates as master regulators of cell metabolism through control of the balance between glycolysis and mitochondrial oxidative phosphorylation in ATP production [25], likely affecting cell phosphate homeostasis [26]. These important features rely on the di(β)phosphate group to enable competition of these molecules with phosphatidylinositol-3,4,5-trisphosphate (PIP_3_) in order to bind to pleckestrin homology domains (PH) [27]. In mammals, generation of knockout mouse models has established the in vivo impacts and significance of IPs pathways [28], while pharmacological modulation of inositol and its pyrophosphate-related pathways have proved to be beneficial in several pathological settings [29,30,31].

**Figure 1:**
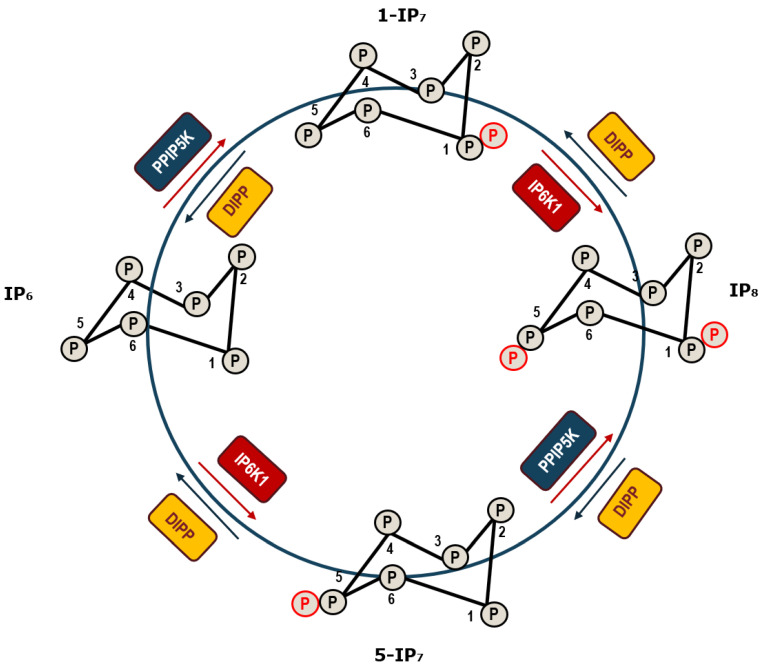
Diagram of the biosynthesis steps by which IP_6_ is sequentially into IPs in mammalian cells. IP_6_, inositol hexakisphosphate; 5-IP_7_, diphosphoinositol pentakisphosphate-5, 5-IP7; DIPP, diphosphoinositol polyphosphate phosphohydrolase; PPIP_5_K, diphosphoinositol pentakisphosphate kinase; IP_6_K, inositol hexakisphosphate kinase.

Diphosphoinositol pentakisphosphate (IP_7_) has been extensively studied and demonstrated to play a pivotal role in pathways related to PP-IP activities [32]. Saiardi et al. showed that IP_7_ physiologically transfers the β-phosphate of the pyrophosphate moiety to several target proteins, implying a major role in protein signaling [33]. IP_7_ intracellular biosynthesis is closely regulated and is catalyzed by two classes of enzymes: inositol hexakisphosphate kinase (IP_6_K, Kcs-1 in yeast) [34,35] and diphosphoinositol pentakisphosphate kinase (PPIP_5_K, Vip1 in yeast) [36], generating two IP_7_ isomers. Thus, these enzymes add a β-phosphate to the pre-existing phosphate at position 5 or 1 on the inositol ring of IP_6_ to generate the 5-IP_7_ or 1-IP_7_ isomer [37,38] (Figure 1).

On the other hand, dephosphorylation of inositol pyrophosphates to IP_6_ or IP_5_ is catalyzed by the enzyme diphosphoinositol polyphosphate phosphohydrolase (DIPP), which exists as five isoforms in mammals, while only a single isoform (Ddp1, diadenosine and diphosphoinositol phosphohydrolase) has been found in yeast [39] (Figure 1). Recently, Vip1 class of enzymes have a pyrophosphatase domain, thus lowering PP-IPs levels and harboring dual functionality [40,41].

This is why PP-IP levels in cells oscillate continuously, being chemically reactive and highly labile, they are a specific target of active cellular phosphatases. IP_7_ is the most abundant (~2–5 μM) in cells, whereas IP_8_ (1,5-bis-diphosphoinositol 2,3,4,6-tetrakisphosphate, 1,5PP-P_4_) is detected at levels 5- to 10-fold lower than those of IP_7_ [42]. However, notwithstanding these relatively low levels, IP_7_ and IP_8_ both play regulatory roles [20]. Since analytical determination of inositol pyrophosphates is a challenging task, instead of a direct estimate, monitoring IP_6_K levels and activity could be a valuable alternative for investigating PP-IP turnover.

The high turnover of PP-IPs shows significant ATP-dependent fluctuations, which operate as an energy-monitoring rheostat [43]. It has therefore been hypothesized that IP_7_ can act as a “metabolic messenger” to coordinate energy flux and signaling pathways, as long as its biosynthesis depends on availability of ATP [19,44].

Indeed, IP_7_ and IP_8_ synthesis both depend closely on ATP availability, since starvation or abridged availability of ATP have been shown to strongly reduce inositol pyrophosphate concentrations in different cell models [45,46]. Conversely, inositol pyrophosphates increase in response to a wide range of physical (thermal [47], osmotic [48]) and energy stressors [49], which ultimately increases ATP availability, ultimately through AMPK modulation. However, this evidence suggests that IP_7_ and IP_8_ behave as “energy sensors,” quite a different concept from the classical “second messenger” initially proposed. It should be underlined that the free energy of hydrolysis of the pyrophosphate moiety is similar to that of the high-energy bond found in ATP [50].

Regarding inositol phosphates, PP-IPs are chiral in nature and can allosterically regulate protein activity through binding to specific domains. Importantly, 5-IP_7_ can compete with phosphatidyl-inositol-3,4,5-trisphosphate (PI-3,4,5-P3, PIP_3_), by specifically binding to pleckstrin homology domains, thus inhibiting PIP_3_-PH-domain interaction [51], as already observed for IP_4_ and IP_6_, albeit with greater affinity [52]. It is noteworthy that 5-IP_7_ synthesized by IP_6_K2 stimulation can bind and activate the protein kinase CK2, thus triggering a number of major biological effects, including apoptosis [27]. Inositol pyrophosphates also regulate the histone deacetylase Rpd3L, a key factor in the regulation of metabolic adaptation to a wide array of stresses [53], thereby affecting gene expression in phosphate starvation, glycolysis, ribosome biogenesis, and environmental stress response pathways [54]. Some inositol phosphates (IP_2_, IP_5_) have already been shown to participate in modulating class 1 histone deacetylases (HDACs) HDAC1 and HDAC3 [55].

## 3. IP_6_Ks: Balance, Activity, and Regulation in Physiological Homeostasis and Cancer

IP_6_Ks have been identified in several organisms [38,56,57]. In mammals, the three isoforms identified [58,59] have distinct sequences that are selectively involved in protein–protein interactions and post-translational modifications [59]. These regions of IP6Ks protein sequence regulate the activity, stability, subcellular distribution, and target proteins of IP_6_Ks [24,33]. The isoforms also differ in tissue expression. In humans, IP_6_K1 is widely expressed, while IP_6_K2 is higher in the breast, thymus, colon, adipose tissue, testis, prostate, and smooth muscle. In heart and skeletal muscle, IP_6_K3 is the most expressed form [60]. The IP_6_Ks belong to the same family of inositol phosphate kinases as IP_3_K (IP_3_-kinase) and IPMK (inositol phosphate multikinase), all characterized by a common PxxxDxKxG motif in the inositol binding region [61]. On the contrary, PPIP_5_K1 and PPIP_5_K2—homologs of the yeast enzyme Vip1—do not belong to the inositol phosphate kinase family, as they have a histidine acid phosphatase-like domain in the C-terminal portion of the protein in addition to the kinase domain [62].

IP_6_Ks can phosphorylate IP_6_ to 5-IP_7_ and IP_5_ to PP-IP_4_ [63]. It is arguable that the relative affinities of a given IP_6_K for IP_6_ over IP_5_ vary in different organisms, from yeast to mammals. For instance, in humans, IP_6_K2 displays a 20-fold higher affinity for IP_6_ than for IP_5_, while IP_6_K1 shows a 5-fold higher K_M_ (concentration of substrates when the reaction reaches half of Vmax) for IP_6_ than for IP_5_ [38].

Furthermore, measurement of IP_6_Ks has advantages with respect to direct quantitation of PP-IPs. Estimation of inositol PP-IPs suffers from a number of problems, including intrinsically higher chemical reactivity and a higher degradation rate, which can be ascribed to the intrinsic acidic phosphatase domain of PPIP_5_K and to the hydrolytic activity exerted by DIPP (diphosphoinositol-phosphate phosphohydrolase) proteins [64]. Indeed, previous studies have been unable to detect a change in PP-IPs in response to biochemical/metabolic stimuli [17], although further investigations have provided compelling evidence in support of this hypothesis [65]. On the other hand, noncatalytic functions of IP_6_K could make tricky the association with PP-IPs signaling. It has also been demonstrated that PP-IPs turn over rapidly (recruiting up to 50% of the IP_6_ pool), depending on chemical (ATP and fluoride) stimulus [16] or during specific cell phase transitions, such as those of the cell cycle [66].

This finding is the hallmark of a substrate cycle involving molecules with high-energy bonds that can play an important role in cell physiology and be targets for cell regulation, as in other metabolic cycles. Some studies have shown that the activity of IP_6_Ks depends on changes in the ATP/ADP ratio [67]. Both IP_7_ and IP_8_ act by limiting ATP synthesis, downregulating glycolysis, and oxidative phosphorylation, and this effect depends remarkably on insulin stimulation [68]. Indeed, when the ATP/ADP ratio decreases, IP_7_ levels are affected negatively, suggesting that IP_6_K activity is significantly downregulated in these situations [57]. Conversely, intracellular ATP levels accumulate in IP_6_K1/IP_6_K2 double knockout cells and in PPIP_5_K-null cells [69,70].

One hypothesis considers the IP_6_K1 isoform as a main sensor of changes in the ATP/ADP ratio [57]. IP_6_Ks have a K_M_ for ATP close to 1 mM, a value within the range of intracellular ATP oscillations [67]. It can be hypothesized that rises in inositol pyrophosphates cooperate with insulin in responding to fluctuations in intracellular ATP levels. The K_M_ for ATP of a wide array of inositol phosphate kinases (IP_3_K, IMPK, IPPK, and PPIP_5_K) usually is in the range between 20 and 100 μM, whereas the K_M_ of IP_6_K is significantly lower (1.0–1.4 K_M_) [37,71].

On the other hand, IP_7_ levels can be efficiently modulated by interfering with PI3K activity. Indeed, restriction of the intracellular inositol phosphates pool, as obtained downstream of PI3K inhibition (by specific PI3K inhibitors like wortmannin and LY294002), reduces IP_7_ and IP_6_K1 levels [57].

The activity of IP_6_K is closely coupled to activation of G protein signaling. G protein-coupled receptor (GPCR) activation through overexpression of G_αq_ fosters phospholipase-C-dependent release of IP_3_ by phosphatidyl-inositol-bisphosphate (PIP_2_) cleavage [72]. In turn, the increased availability of IP_3_ provides the substrate for inositol kinases to produce a plethora of inositol phosphates (chiefly, IP_6_ and IP_5_) and inositol pyrophosphates (PP-IPs). Overexpression of IP_6_K only results in a minimal increase in PP-IPs, even in the presence of high levels of IP_5_ and IP_6_, while when IP_6_K is overexpressed together with GPCR activation, a significantly increased release of PP-IPs has been recorded [72]. These findings suggest a cooperative network linking GPCR and IP_6_Ks, which can tune inositol metabolism by acting as an “IPK-dependent IP code” [72]. This hypothesis has contributed to a revision of the role traditionally attributed to IP_6_. It is widely agreed that inositol hexakisphosphate displays a bewildering number of physiological and pharmacological activities [10]. However, the IPK-dependent IP code hypothesis may substantiate the suggestion made 20 years ago by Shears [12] who proposed that the critical importance of IP_6_ may depend on being a tipping point between IP_3_ and the successive generation of IPs. Indeed, increasing evidence in recent years has provided sound confirmation that it is the further phosphorylation of IP_6_ to IPs that yields physiologically active metabolites [73]. Any factor that potentiates IP_3_ release through phospholipase-C activation is likely to reduce PIP_2_ levels while promoting inositol phosphokinase (IP_6_K) activity. Accordingly, phospholipase-C and IP_6_K both seem to play a potentially critical role in several biological pathways.

A recent paper displayed a new biosynthetic route that can originate directly from the conversion of glucose-6-phosphate (G6P) to IP_1_. Starting from this point, during phosphate starvation, a “soluble” lipid-independent metabolic pathway is triggered by ITPK1, a kinase, leading to IP_6-7-8_ synthesis [74]

Furthermore, IP_6_Ks are also involved in tailoring protein activities by modulating scaffold/protein-based interactions, which usually do not require IP_6_K-related catalytic activity. Binding of IP_6_K1 to glycogen synthase kinase (GSK3) [75], interaction of IP_6_K2 with TNF receptor-associated factor-2 (TRAF2) [76], and binding of IP_6_K3 to spectrin and adducin [77] are all processes related to such interactions.

### 3.1. IP_6_K1

IP_6_K1 has been implicated in biological processes, such as energy metabolism, insulin signaling, trafficking, chromatin remodeling, cell migration, cancer metastasis, and neutrophil functions.

Recent studies suggest that in IP_6_K1-KO mice models, IP_6_K1 suppression increases energy expenditure by stimulating the protein kinase AMPK [27,78]. AMPK and Akt are significantly modulated under insulin stimulation [79]. IP_6_K1 could modulate AMPK and Akt activities by interfering with insulin release. The link between IP_6_K1 and Akt merits detailed discussion. Akt resides in the cytosol in an inactive conformation and translocases to the plasma membrane after cell stimulation. The Akt pleckestrin homology domain has a high affinity for PIP_3_, which promotes Akt translocation to the membrane [80]. The Akt/PI3K interaction causes conformational changes and subsequent PDK1-dependent phosphorylation at the Thr^308^ kinase domain. However, full activation requires a further phosphorylation at S473, catalyzed by several enzymes, including PDK2 and ILK. IP_7_ competitively binds to the PH domain, thus preventing its phosphorylation and activation by PDK1. Notably, IP_7_ strongly inhibits Akt activation, with an IC50 of 20 nM, close to the Kd (35 nM) displayed by PIP_3_ in respect to the PH domain of Akt [81]. IP_6_K1 knockout leads to increased PDK1-dependent Akt activation, determining a plethora of biochemical consequences for metabolic regulation, not yet well investigated. Indeed, after glucose stimulation and subsequent increase in the ATP/ADP ratio, a significant increase in IP_7_ was observed. In detail, IP_7_ production by IP_6_K1 inhibits the stimulatory effect of IP_6_ on AMPK. The response of IP_7_ to the increase in ATP/ADP ratio occurs a few minutes (10–30) after the stimulus. In turn, IP_7_ associates with the Akt PH domain, preventing interaction with PIP_3_ and therefore reducing Akt membrane translocation and consequent insulin-stimulated glucose uptake. This mechanism involves feedback, whereby increased availability of ATP drives the system to inhibit glucose uptake by modulating insulin transduction by blocking Akt membrane recruitment [82,83,84]. This regulation may also be indirectly affected by IP_7_-promoted nuclear localization of LKB1. Nuclear transfer of LKB reduces LKB cytosolic activity, thus hindering AMPK phosphorylation and activation [85]. It is worth noting that RNAi silencing of IP_6_K1 blocks IP_7_ and insulin release after glucose stimulation. In IP_6_K1-KO models, changes in the intracellular IP_6_/IP_7_ ratio increase AMPK activation [86]. Conversely, Akt signaling is significantly increased, leading to a decrease in GSK3b phosphorylation, and augmented protein translation. Reduction in GSK3b phosphorylation increases its catalytic activity and is likely be followed by a surge in adipogenesis and diminished glycogen levels [87]. Indeed, after insulin stimulation, IP_7_ decreases (from 33% to 60%) in IP_6_K1 knockout hepatocytes, whereas Akt and GSK3β increase, improving glucose tolerance, presumably due to a decrease in hepatic glucose production [26]. Conversely, overexpression of IP_6_K1 finally impairs insulin-signaling transduction, whereas IP_6_K1 silencing may lead to insulin hypersensitivity, as observed in IP_6_K1 KO mice. As proof of concept, a number of animal models of insulin hypersensitivity share the common biochemical signature of an increased tier of Akt activation and translocation [88]. Furthermore, in mouse embryo fibroblasts (MEFs), IP_6_K1-induced energy expenditure inhibition leads to reduction of glycolysis via IP_7_-mediated destabilization of the interaction between the transcriptional activators of glycolytic genes (GCR1 and GCR2) [25].

Although IP_6_K2 proves sensitive to ATP/ADP fluctuations and may induce IP_7_ synthesis, it is unlikely that it could act as a sensor of energy requirements, as does IP_6_K. This apparent conundrum can be explained if we consider the cell compartmentalization of IP_6_K. In fact, while IP_6_K1 is usually found in the cytosol and nucleus, IP_6_K2 is almost all in the nucleus [89].

However, studies performed with pancreatic β cells have shown that an optimal level of IP_7_ generated by IP_6_K1 is critical for proper insulin exocytosis [90] as confirmed—by studies with—IP6K1-KO mice [91]. In fact, IP_7_ reduces insulin-dependent activation of Akt by regulating insulin secretion and pleiotropic signaling, as observed in type-2 diabetes (T2D) [82]. Accordingly, IP_6_K1 deletion may indirectly hinder insulin release by interfering with the regulation of Ca^2+^-dependent activator protein, a protein necessary to enable insulin release in response to Ca^2+^ stimulation [83].

These findings have prompted a contrary hypothesis. Chakraborty et al. [26] postulate that selective inhibitors of IP_6_K1 have therapeutic potential for treating type-2 diabetes associated with obesity and insulin resistance, whereas Bhandari [91] considers lower fasting serum insulin in IP_6_K1 knockout mice to be evidence of a mandatory activity of IP_6_K1 in enhancing insulin release from pancreas β cells. However, IP_6_K1 knockout in mice does not lead to diabetes, although plasma insulin is reduced [26]. One can posit that insulin reduction after IP_6_K1 silencing can instead be interpreted as a sign of increased sensitivity to insulin secretion: reduced IP_7_ levels by IP_6_K1 inhibition enhance cell responsiveness to even lower levels of insulin, so as to maintain an appropriate rate of glucose uptake [92].

Finally, it is even more likely that the effects of IP_6_K1 on insulin are a secondary epiphenomenon, while ATP/ADP fluctuations are preferentially the master regulator of IP_7_ release, as mentioned above [43]. Furthermore, it would be worthwhile investigating the relationships between IP_6_K1/IP_7_ and mitochondrial activity. Insulin resistance is associated with mitochondrial dysfunction [93], while improvement of mitochondrial biogenesis may reduce insulin resistance. Interestingly, an increase in IP_7_ levels is associated with abnormal biogenesis and mitochondrial dysfunction, while selective IP_6_K1 inhibition restores mitochondrial function and insulin sensitivity [94]. It is therefore tempting to speculate that IP_6_K1 overexpression could be associated with dysfunctional mitochondrial activity, including ATP production during the oxidative catabolism of glucose. An unbalanced ATP/ADP ratio could in turn explain the consequent abnormalities in insulin responsiveness. Further studies are required to shed light on this intricate matter.

Until now, it has been deemed that PP-IPs modulate protein function in two ways: (a) by direct binding to a target protein or (b) by enabling a post-translational modification through pyrophosphorylation. For instance, transfer of the phosphate group from IP_7_ to phosphoserine has been observed in RNA-polymerases [95], suggesting that PP-Ips can act at post-translational level, slightly modulating RNA-dependent processes.

However, there is increasing evidence that a number of IP_6_K1-related effects on cell biochemistry may be mediated by regulation of gene expression via chromatin remodeling, since the nuclear localization of IP_6_K1 is shown to modify DNA methylation modulating DNMTs activity [59].

Eukaryotic DNA is packaged in a complex, intertwined manner and closely modulates genome transcription. Coordinated remodeling of chromatin at selected places enables transcription at specific sites, through modulation of DNA and histone methylation. It is noteworthy that nuclear-localized IP_6_K1 participates in modulating these processes.

For instance, IP_6_K1 enhances DNA methylation in a catalytic activity-dependent manner and subsequently inhibits transcription of the inositol biosynthesis gene in mammals (inhibition of the *ISYNA1* gene) [96]. In yeast, inositol biosynthesis is transcriptionally regulated by *INO1*, the gene encoding 3-phosphate synthase, the enzyme that promotes inositol-phosphate synthesis from glucose-6-phosphate (G6P). In turn, INO1 expression is controlled by the transcriptional repressor Opi1 in response to inositol and phosphatidic acid (PA) levels [97]. Opi1 is stabilized by physically interacting with PA on the endoplasmic reticulum membrane. In the presence of low levels of *myo*-inositol, PA values increase as they are spared from being utilized for phosphatidylinositol synthesis. Thus, Opi1 remains in the endoplasmic reticulum, physically bound to PA. On the contrary, high inositol content in the cytoplasm leads to an increase in phosphatidyl-inositol synthesis, thus freeing Opi1 from PA binding. Opi1 can then translocate into the nucleus, where it represses *INO1* transcription, resulting in decreased inositol synthesis. However, inositol biosynthesis requires the participation of Kcs enzymes—the yeast homolog of IP_6_Ks—and increases PP-IP production [98]. Surprisingly, a completely different picture is observed in mammalian cells. The gene homologous to *INO1* in metazoan cells is *ISYNA1*, which is dramatically upregulated by knock-out of IP_6_K1. IP_6_K1 behaves like the yeast repressor Opi1 as it binds to PA in the cytosol, then translocating into the nucleus and acting as a negative regulator of *ISYNA1*. Conversely, *ISYNA1* upregulation in IP_6_K1-KO cells is most likely due to reduction of DNA methylation [96]. This effect could involve a number of mechanisms, including reduced recruitment of transcription factors to the promoter region of *ISYNA1* or altered assembly of the transcription complex. In contrast to positive regulation of *INO1* in yeast, PP-IPs and IP_6_K1 negatively regulate *ISYNA1* transcription. Thus, we can hypothesize a negative feedback in which IP_7_ is able to regulate the triggering of the soluble pathway [74] by ISYNA1 inhibition and thus the synthesis of IP_6_ and IP_7_ itself.

In MEFs, IP_6_K1-induced histone methylation seems to involve histone lysine demethylase JMJD2C interaction [99]. Reducing IP_6_K1 levels by RNAi or using mouse embryo fibroblasts derived from IP_6_K1 KO mice results in decreased IP_7_ concentrations that translate epigenetically into reduced levels of trimethyl-histone H3 lysine 9 (H3K9me3) and increased levels of acetyl-H3K9. Binding with IP_6_K1 causes JMJD2C to dissociate from chromatin, hence increasing H3K9me3 levels and blocking the transcription process of JMJD2C target genes [99].

Moreover, without exerting any catalytic activity, IP_6_K1 can form a ternary complex with COP9 signalosome (CSN) and Cullin-RING ubiquitin ligase (CRL4). Dissociation of IP_6_K1 and subsequent generation of IP_7_ under UV exposure activates CRL4, which in turn promotes substrate ubiquitylation and ultimately regulates nucleotide excision repair and cell death [100]. The negatively charged phosphate of IP_7_ interacts with a positively charged canyon surface of CRL4, eliciting conformational changes, but only after IP_6_K1 has dissociated from the complex. This mechanism seems to be specific to UV-dependent DNA damage, since homologous repair activity in mouse embryo fibroblasts exposed to hydroxyurea, responsible for double-strand DNA breaks, is undetectable upon IP_6_K1 deletion [91]. This finding suggests that IP_6_K1 noncatalytic activity is required to inhibit CRL4, while IP_6_K1 enzyme activity (leading to increased IP_7_ release) is also necessary for proper CRL4 activation.

IP_6_K activities are not limited to energy metabolism and modulation of gene expression, as IP_6_K1/IP_7_ levels affect vesicle trafficking through pyrophosphorylation of cytoskeletal proteins.

IP_6_K1 regulates neuroexocytosis through enzyme-dependent and independent mechanisms. Inactive and active IP_6_K1 catalytic forms inhibit the nucleotide exchange factor GRAB, by competing for binding to Rab3A. As GRAB/Rab3A complexes are required to trigger exocytosis from axons, IP_6_K1/IP_7_ reduces neuroexocytosis in PC12 cells stimulated with Ca^2+^ [101]. Similarly, by interacting with the C2-domain of synaptotagmin 1 (SYT1), a critical mediator of fast and calcium-dependent neurotransmitter release, IP_6_K1/IP_7_ suppresses Ca^2+^-mediated neuroexocytosis in PC12 and in hippocampal neuronal cells [102], as already noticed with others inositol phosphates (IP_4_ and IP_6_) [103].

In MEFs, IP_7_ inhibits kinesin-induced exocytosis but facilitates dynein-mediated trafficking, through IP_7_-mediated pyrophosphorylation of Ser51, which lies in close proximity to the core p150^Glued^-binding region of dynein [104]. Dynein phosphorylation stabilizes an ordered conformation of the protein, thus facilitating recruitment of multiple dynein motors; this would counteract the effect of kinesin and thus organelle movement towards the plus end of microtubules [105]. Expression of catalytically active but not inactive IP_6_K1 reverses these defects, suggesting a role of inositol pyrophosphates in these processes. In metazoan cells, short-range vesicle displacement—inside or outside the cell—is an actin/myosin-dependent process. Instead, long-range transport occurs along cytoskeletal microtubules and is mostly driven by kinesins, which move vesicles towards the plus-end of microtubules, behind the cell membrane, and dynein, which carries vesicles to the minus-end of microtubules, close to the nucleus [106]. Interestingly, PP-IPs have been shown to negatively regulate the interaction of the kinesin motor Kif3A with the adaptor protein 3 (AP3), thus limiting exocytosis [107]. Furthermore, yeasts lacking PP-IPs show altered vacuole morphology due to defective endosomal sorting [108]. Moreover, the transfer of a high-energy β-phosphate from IP_7_ to a phosphorylated serine residue to form pyro-phosphoserine can significantly modify protein–protein interactions [24]. Since these amino acid residues are usually expressed by membrane proteins, it is readily argued that IP_7_ can modulate membrane reactivity and trafficking just by modifying the phosphorylation status of these key membrane-bound complexes.

Overall, by confirming how IP_6_K1 and PP-IPs are intertwined in actin–myosin and microtubule-dependent kinesin-driven processes, these studies suggest that IP_6_Ks and their metabolic products can also sustain an appreciable role in membrane trafficking and cytoskeleton-dependent activities. Indeed, IP_6_K1 can participate in cytoskeleton remodeling by interfering with different biochemical pathways—including the PI3K/Akt cascade—and with a number of cytoskeletal proteins, such as FAK and paxillin. High levels of IP_6_K1/IP_7_ are indeed crucial for regulating cell migration in physiological and pathological processes. In brain development, impairment of IP_6_K1 activity decreases neuronal migration. IP_6_K1 binds to α-actinin, which is associated with FAK, that together with α-actinin constitutes the focal adhesion complex. Remarkably, IP_7_ enhances autophosphorylation of FAK, which in turn augments neuronal migration [109]. IP_6_K1 also contributes to regulation of cytosolic distribution and the architecture of stress fibers, a critical component in determining cell shape and function [109].

However, IP_6_K1 activation/depletion may lead to significantly different issues in relation to tissue-dependence. For instance, IP_6_K1 reduction favors phosphorylation-based Akt activation while increasing neutrophil superoxide production and bactericidal activity, without altering cell adhesion and migration [110]. Neutrophils from IP_6_K1 KO mice accumulate in the lungs and probably contribute to chronic obstructive pulmonary disease (COPD) [111], while when stimulated with IP_6_K1, IP_7_-mediated Akt inhibition enables neutrophil death and protects against COPD [112]. Finally, IP_6_K1 depletion negatively affects motility and phagocytosis in macrophages.

Unfortunately, the role of IP_6_K1 in cancer motility and invasiveness has received little or no attention, despite the fact that some reports have identified it as a target for reducing migration and invasion in several types of cancer [113]. Suppression of IP_6_K1 significantly reduces the migrating capacity of MEFs and this function depends on its ability to synthesize inositol pyrophosphates, while depletion of IP_6_K1 in HeLa and HCT116 cells is reported to result in a significant decrease in chemotactic migration towards serum-rich medium over a period of 24 h [113]. Since the tumorigenic and metastatic potential of cells depends on their migratory and invasive properties, which require dramatic reorganization of the actin cytoskeleton [114], the fact that IP_6_K1 can actively participate in cytoskeleton remodeling is of utmost importance. IP_6_K1 is indeed involved in adhesion-dependent signaling and the resulting cytoskeletal remodeling that controls cell spreading. It has been observed that IP_6_K1 acts upstream of integrin-growth factor synergies by promoting FAK phosphorylation [104]. IP_6_K1 silencing has been found to interfere with integrin-mediated signaling events, thus leading to reduced activation of FAK and paxillin, two intermediate keys of cytoskeleton remodeling. Phosphorylation of FAK and paxillin (a scaffold protein that is phosphorylated by FAK and recruits several proteins required for cytoskeletal reorganization during cell spreading) was significantly inhibited in IP_6_K-null cells (MEFs). Defects in invasiveness and in the migrating capacity of MEFs were completely restored on expression of active but not inactive IP_6_K1, suggesting that inositol pyrophosphate synthesis is required to support cell migration [113]. Modulation of cytoskeleton remodeling is a property shared by a number of inositol phosphates and by inositol itself [115]. Inositol pyrophosphates synthesized by IP_6_K1 could act in a similar manner, influencing the activity of transcription regulatory proteins or even gene expression (through epigenetic mechanisms such as those related to histone modulation), which are coupled with cytoskeleton rearrangement.

Broadening the spectrum, myo-inositol *per se* has been shown to dramatically modulate cancer migration and invasiveness [115]. This effect is in part mediated by increasing ISYNA1 activity [116]. We can therefore surmise that myo-inositol also exerts an anticarcinogenic role by modulating IP_6_Ks through complex feedback involving some critical enzymic and genomic steps [117]. It can also be hypothesized that hyperactivity of IP_6_K1 and the consequent increase in IP_7_ synthesis will deplete the IP_6_ intracellular pool, as IP_6_ is the major intermediate in IP_7_ production. This is indeed the case, as IP_6_K1 acts as an IP_6_-dephosphorylating enzyme, thus depleting the IP_6_ cellular pool [67]. Because IP_6_ displays a wide array of anticancer functions inside the cell, it can be surmised that reduction of IP_6_ stores downstream of IP_6_K activation may foster a number of carcinogenesis-related pathways [118].

Suppression of IP_6_K1 may be considered an attractive option in integrated anticancer strategy. However, it has been objected that complete suppression could have detrimental effects, since IP_6_K1 has been shown to play an important role in maintaining genomic integrity by promoting DNA repair [119] and favoring nucleotide excision repair [100], two key pathways, impairment of which could enhance the spontaneous development of tumors. However, IP_6_K1 deletion does not imply complete disappearance of IP_7_ from cells, as only a 70–80% reduction has so far been recorded in IP_6_K1 null mice [91], while IP_6_K2 activity could account for the remaining 20–30% of IP_7_ synthesis, as documented in studies with MEFs in which the IP_6_K2 gene has been deleted [27]. In other cells, like HCT116 cells, the respective contribution of IP_6_K1 and IP_6_K2 to IP_7_ synthesis may even be different [22,120]. In any case, these findings suggest that even with complete silencing of one of the two IPKs, the other can successfully ensure minimal, albeit physiologically significant, levels of PP-IP_5_, high enough to avoid the risk of cancerous transformation. It is likely that a proper balance in the activity of IP_6_Ks is required to modulate cell motility, preventing cancer transformation; a valid pharmacological endeavor would aim at modulating, rather than abolishing, IP_6_K-dependent IP_7_ synthesis.

### 3.2. IP_6_K2

A number of studies suggest an essential role for IP_6_K2 in cell death, migration, cancer metastasis, and progression. IP_6_K2 activity sensitizes a number of cancer cells, including OVCAR3, HeLa, HEK293, PC12, and HL60, to apoptosis [121,122,123,124]. Deletion of IP_6_K2 prevents apoptotic consequences of γ-irradiation or β-interferon addition to ovarian cancer cells, while overexpression of IP_6_K2 significantly raises cell death rate under the same conditions [122]. Overexpression of IP_6_K2 augments the cytotoxic effects of many cell stressors, whereas transfection with a dominant negative IP_6_K2 decreases cell death. It is noteworthy that the apoptosis surge is associated with increased synthesis of IP_7_ and transfer of IP_6_K2 from nuclei to mitochondria, while no changes are recorded in the intracellular localization of the other IP_6_K isoforms [121]. In detail, IP_6_K2 directly mediates IFNβ-induced apoptosis [121] by enzymically regulating p53 activity and by increasing expression of the Apo2L/TRAIL ligand that initiates apoptosis through death-receptor signaling. Namely, HSP90 physiologically binds IP_6_K2 and inhibits its catalytic activity. By interfering with HSP-IP_6_K2 binding, HSP90 fosters IP_6_K2 activation that ultimately leads to increased cell apoptosis [21]. Nuclear localization of IP_6_K2, promoted by interaction with HSP90, is a mandatory step for establishing proper IP_6_K2-p53 binding. [22]. Indeed, IP_6_K2 has been demonstrated to directly modulate p53-dependent apoptosis. Gene disruption of IP_6_K2 in colorectal cancer cells selectively impairs p53-mediated cell death and favors cell cycle arrest [22]. This interaction suppresses phosphorylation of the cell cycle arrest regulator (p21) and its transcription, while enhancing p53-mediated apoptosis [23]. This implies that IP_6_K2 acts as a switching factor, driving p53 activity towards apoptosis rather than cell cycle arrest. It should be noted that although IP_6_K2 regulates p53 by direct binding, its catalytic activity generating IP_7_ is essential for its influence on p53 signaling. It has also been observed that IP_6_K2 can promote apoptosis independently of its enzyme activity. By interacting with TRAF2, IP_6_K2 interferes with apoptosis and nuclear factor kappa β (NF-kβ) signaling, thus affecting the release of tumor necrosis factor α (TNFα) [33]. The proapoptotic activity of IP_6_K2 is successfully antagonized by heat-shock proteins (HSPs). Overall, these findings suggest that IP_6_K2 actively participates in the regulation of the Apo2L/TRAIL cell death pathway. Moreover, PP-IPs modulate cell death and telomere length in yeast by antagonizing the homolog of ataxia telangiectasia mutated (ATM) kinase, a regulator of the DNA damage response and apoptosis in mammals [125].

As strong as IP_6_K2-mediated apoptosis may be, IP_6_K2 participation in the regulation of such functions through its nuclear [126], mitochondrial [122], and cytosolic [123,127] localization requires further investigation.

As observed in IP_6_K1-KO models, IP_6_K2-KO, too, reduces cell–cell adhesion, growth, spreading, metastasis, and FAK phosphorylation in cancer cells. The molecular mechanisms so far proposed include LKB1 sequestering in the nucleus and inhibition of cytosolic phosphatase activation, and consequently, FAK dephosphorylation [125]. Remarkably, IP_6_K1 and IP_6_K2 both favor sequestering of LKB into the nucleus in an inactive form [85,127].

The tumor suppressor LKB1 is credited with inhibiting FAK activation [128] and enhancing E-cadherin expression [129], thus inhibiting motility and invasiveness. These findings strongly suggest that LKB1 plays a critical role in controlling the balance between cell–cell and cell–matrix adhesion. In addition, by modulating AMPK activity, LKB1 interferes with a number of critical metabolic processes [130]. Interaction with two subunits of the heterotrimeric holoenzyme (STRAD and Mo25) in the cytosol leads to phosphorylation of LKB1 at serine-428 and then activation by PKCδ [131]. This finding is worth mentioning as it suggests that IP_6_K2/IP_7_ can fine tune the activity of “constitutive” kinases, like PKCδ and CK2 [27], as previously indicated.

On the contrary, LKB sequestration in the nucleus in an unphosphorylated form prevents its activation [131]. IP_6_K2 decreases phosphorylation of cytosolic LKB1 in HCT116 and HEK293 cells and establishes a complex with LKB1 that translocases into the nucleus in an inactive form [127]. The enzymically active form of IP_6_K2 is mandatory to inhibit LKB1, suggesting that IP_7_ synthesis participates in LKB1 sequestration. As a result, inactivation of LKB1 by IP_6_K2/IP_7_ shifts the balance between cell–cell and cell–matrix adhesion to favor focal adhesion while weakening cell–cell adhesion, finally leading to enhanced cell migration/invasiveness. Furthermore, IP_6_K2 is downregulated when epithelial–mesenchymal transition (EMT) is inhibited or mesenchymal–epithelial transition (MET) is triggered [132]. These findings suggest that IP_6_K2 downregulation is required to preserve the epithelial phenotype and to antagonize the emergence of invasive-migrating mesenchymal-like phenotypes.

Indeed, a number of results have clearly established that deletion of IP_6_K1 or IP_6_K2 reduces cell migration, while IP_6_K2-KO, quite paradoxically, reduces tumor volume [133]. IP_6_K2-KO cells display almost total loss of IP_8_ levels, whereas only a small decrease in IP_8_ levels was recorded in IP_6_K1-KO [23,113]. It is tempting to speculate that persistent IP_7_ synthesis, even at a lower rate, is mandatory for apoptosis, as previously suggested. However, somewhat paradoxically, complete suppression of IP_6_K2 enhances development of carcinoma of the gastrointestinal tract in mice [134], probably because IP_6_K2-dependent pyrophosphate synthesis may in turn activate p53 and protein kinase CK2, thus promoting apoptosis [2]. In IP_6_K2 knockout mice, a substantial increase in tumorigenesis in response to 4-nitroquinoline-1-oxide, a UV-mimetic carcinogen, has been observed [135]. These findings provide indirect confirmation of the link between IP_6_K2 and p53, as p53-mediated apoptosis is required for apoptosis induced by UV-mimetic factors. However, unlike p53 knockouts models, the IP_6_K2 mutants do not develop spontaneous tumors. This apparently odd behavior suggests that IP_6_K2 may only influence p53 proapoptotic activity when the system is exposed to a carcinogen stressor but does not directly entail “spontaneous” carcinogenesis.

In ovarian carcinoma cells, IP_6_K2 deletion confers protection against interferon alpha (IFNα)-induced cell death, whereas overexpression of IP_6_K2 enhances the apoptosis rate promoted by IFNα and/or γ-irradiation [136]. Yet some controversial results have also been reported, since under estradiol stimulation, β-catenin-induced oncogenesis significantly increases IP_6_K2 gene expression downstream of the Wnt/β-catenin signaling pathway [137]. Overexpression of IP_6_K2 presumably leads to increased pyrophosphate synthesis, reducing cell levels of IP_6_, which may in turn contribute to the transformed phenotype. On the other hand, suppression of IP_6_K1 confers protection against tumors experimentally induced with carcinogens [138].

Although these findings are still preliminary, they suggest that IP_6_K1 and IP_6_K2 can exert opposite effects in carcinogenesis. It is also likely that the effects of IP_6_K2 on cancer cells are disjointed, i.e., IP_6_K2 probably enhances apoptosis while increasing the acquisition of an invading/migrating phenotype. IP_6_K2 may, therefore, act as a tumor suppressor in the initiation stage but contribute to metastatic spread by enacting EMT at later stages. It is worth underlining that similar dual roles have been observed for TGF-β1 [139].

### 3.3. IP_6_K3

IP_6_K3 is highly expressed in mouse and human myotubes and muscle [77]. Its physiological role is relatively unexplored. High levels of expression have been detected in the brain. Purkinje cells regulate motor learning and coordination, and IP_6_K3 deletion alters these functions. Abnormalities in cell size and spine density are detected, perturbed by dysfunctional IP_6_K3 binding of adducin and spectrin, two cytoskeletal proteins involved in the morphogenesis of dendritic trees [77]. Regarding other IP_6_Ks, IP_6_K3 seems to participate somehow in glucose metabolism. Indeed, IP_6_K3-null mice exhibit lower blood glucose and reduced insulin levels, associated with increased plasma lactate levels. These findings suggest that downregulation or suppression of IP_6_K3 can enhance glycolysis. However, IP_6_K3 suppression is followed by a significant reduction in pyruvate dehydrogenase kinase-4 (PDK4) [140]. Since PDK4 depresses glucose oxidation by inhibiting conversion of pyruvate to acetyl coenzyme A (acetyl-CoA), it is paradoxical that IP_6_K3 suppression does not lead to an increase in glucose oxidation.

## 4. Future Perspectives

**Figure 2:**
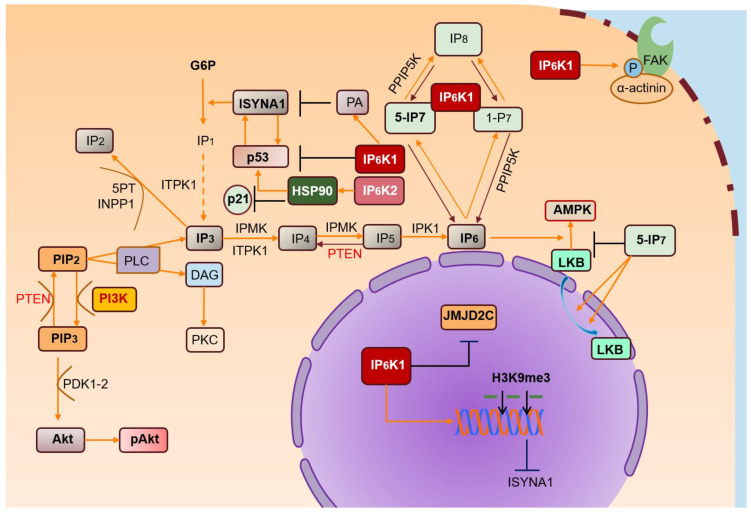
IP_6_Ks and their pathways. IP_3_ is metabolized in many inositol polyphosphates, of which IP_6_ is the most abundant. IP_6_Ks produce IPs (IP_7_) starting from IP_6_. IP_6_K/IP_7_ levels are crucial for regulating various biological processes. IP_6_K1 binds α-actinin localized at focal adhesions, promoting its phosphorylation by FAK and regulating cell migration. IP_6_-stimulated AMPK activation is inhibited by high levels of IP_7_, reducing cytosolic localization of LKB. High PA levels promote nuclear IP_6_K1 translocation, inhibiting ISYNA1, and consequently, de novo biosynthesis of myo-inositol. Nuclear IP_6_K1 interacts with JMJD2C and induces its dissociation from chromatin, increasing H3K9me3 levels and inhibiting transcription of target genes. Likewise, IP_6_K2 may localize in the nucleus, downstream of its interaction with HSP90. In turn, nuclear IP_6_K2 localization promotes binding to p53, suppressing p21 activation and transcription.

Growing interest focused on IPs has shed light on their biological functions and corresponding deregulation issues. Among IPs, IP_7_ plays a significant role in cell metabolic balance, ATP production, and phosphate homeostasis. From these studies, IP_6_Ks emerge as key regulators of IP_7_ intracellular levels in physiological and pathological processes (Figure 2).

Interest in the development of molecular factors that can (selectively) interrogate and manipulate the cell actions of inositol pyrophosphates, especially by modulating IP_6_Ks and PPIP_5_Ks, is gaining momentum [136]. Targeting these pathways could be helpful in certain diseases but also potentially dangerous. For example, knockout experiments on IP_6_Ks highlighted a worse situation in mice, sensitizing the animals to chemical tumorigenesis [135], lung inflammation [112], and loss of motor learning, coordination, and fitness [77,141]. It is therefore crucial to determine whether pharmacological inhibition of IP_6_Ks is safe enough to pursue clinical investigations.

Studies based on gene deletion assays are unlikely to provide useful data, since more than 900 genes are altered by deletion of IP_6_K homolog (Kcs1) in *S. cerevisiae* [54]. The range of this genetic penetration probably highlights the functional polyvalence of IP_6_Ks, which presumably have both catalytic and scaffolding functions, as already demonstrated for inositol pentakisphosphate kinase [142] and inositol polyphosphate multikinase [143]. A more promising approach may focus on specific cell-permeant inhibitors of PP-IPs or on “physiological” modulators of IP_6_Ks, an approach that at least in principle would not be flawed by secondary genetic changes or interference with IP_6_K scaffolding functions.

The compound N2-(m-trifluorobenzyl)N6-(p-nitrobenzyl)purine (TNP) has been shown to bind specifically to IP_6_Ks by competing with ATP for the same binding site. As a result, TNP reduces IP_7_ levels by inhibiting the kinase and phosphatase activities of IP_6_Ks. Within 2 h of treating various cell types with 10–30 μM TNP, levels of IP_7_ fell by 60–90% [67,144], and IP_8_ synthesis was also significantly reduced [145]. As expected, IP_6_ levels increased proportionally by as much as 40%.

TNP does not efficiently cross the blood–brain and blood–testis barriers. In fact, chronic TNP administration (15 weeks, 10 mg/kg/day) in mice does not lead to neuronal or reproductive abnormalities [146]. However, TNP could interfere with the metabolism of other drugs by inducing modifications in drug signaling or increasing Ca^2+^ and Zn^2+^ levels [147].

TNP inhibitory activity discriminates between IP_6_Ks and other inositol phosphate kinases (IPMKs and IP_3_Ks). The catalytic site of the IP_6_K family is structurally related to that of IPMKs and IP_3_Ks, though IP_6_Ks have around 100-fold lower affinity for ATP than do the latter [144]. Higher TNP values are therefore required to efficiently neutralize IP_3_K (IC50 0.47 μM for IP_6_Ks versus 18 μM for IP_3_K). However, TNP displays some off-target effects, including ERK phosphorylation, which in principle is not mediated by IP_6_Ks. The use of TNP to investigate the intracellular functions of IP_6_Ks is therefore debatable. To minimize undesirable effects, it could be useful to develop safe and selective inhibitors of IP_6_K isoforms for investigating the specific role sustained by the different IP_6_K isoforms.

Regarding carcinogenesis, IP_6_K1 and IP_6_K2 activities presumably drive cells and tissues towards opposite outcomes. As previously reported, IP_6_K1 joins in Akt signaling, and its knockout decreases IP_7_ synthesis, resulting in enhanced PDK-dependent phosphorylation of Akt activation. Hyperactivation of Akt (∼10- to 50-fold) [148,149] is known to enable tumorigenesis [150]. However, IP_6_K1-KO is only associated with a minimal increase in Akt activation in mice [27], insufficient to enact neoplastic development [27]. Indeed, it has been reported that deletion of IP_6_K1 protects against chemical tumorigenesis and metastasis [113], although the mechanisms underlying the effect are still unknown. Instead, IP_6_K2-KO sensitizes to chemical tumorigenesis and probably increases the occurrence of spontaneous cancer [138].

The toxicity profiles of IP_6_K inhibitors are likely to make them unattractive. Blood–brain barrier and blood–testis barrier impermeable IP_6_K1 inhibitors with metabolic stability and few side effects could be beneficial in cancer treatments. Welcome developments would be the repurposing of existing drugs or the discovery of natural molecules with such characteristics.

Acronyms: 1-IP_7_ (1-diphospho-2,3,4,5,6-pentakisphosphate); 5-IP_7_ (5-diphospho-1,2,3,4,6-pentakisphosphate); Akt (protein kinase B); AMPK (5’ AMP-activated protein kinase); DAG (diacylglycerol); FAK (focal adhesion kinase); H3K9me3 (histone 3 lysine 9 trimethylation); IP_2_ (inositol-2-phosphate); IP_3_ (inositol-3-phosphate); IP_4_ (inositol-4-phosphate); IP_5_ (inositol-5-phosphate); IP_6_ (inositol-hexakisphosphate or phytic acid); IP_6_K1 and IP_6_K2 (inositol hexakisphosphate kinase 1/2); IPK1 (inositol-pentakisphosphate 2-kinase); IPMK (inositol polyphosphate multikinase); ISYNA1 (d-3-myoinositol-phosphate synthase); JMJD2C (Jumonji domain-containing protein 2C); LKB (liver kinase B1); P (phosphate group); PA (phosphatidic acid); PI3K (phosphatidylinositol 3-kinase); PIP_2_ (phosphatidyl-inositol-4,5-biphosphate); PIP_3_ (phosphatidylinositol-3-phosphate); PKC (protein kinase C); PLC (phospholipase C); PPIP_5_K (inositol hexakisphosphate and diphosphoinositol-pentakisphosphate kinase); PTEN (phosphatase and tensin homolog); P_8_ (1,5-bis-diphosphoinositol 2,3,4,6-tetrakisphosphate); G6P (glucose-6-phosphate); IP_1_ (inositol-1-phosphate, *myo*-Inositol); ITPK1 (inositol-tetrakisphosphate 1 kinase).
